# Perception and experiences of having children and contraceptive use among women in low-income groups: a qualitative study

**DOI:** 10.1007/s00404-025-08058-8

**Published:** 2025-05-28

**Authors:** Fatma Başaran, Pınar Duru

**Affiliations:** 1 Faculty of Health Sciences, Department of Midwifery, Ağrı İbrahim Çeçen University, Ağrı, Türkiye; 2 Faculty of Health Sciences, Department of Public Health Nursing, Eskisehir Osmangazi University, Eskisehir, Türkiye

**Keywords:** Having children, Contraceptive method, Perception, Experience, Women, Qualitative research

## Abstract

**Background:**

Low-income level has great importance on women’s reproductive health issues such as childbearing and contraceptive use.

**Aim:**

This study aims to examine in depth the perceptions and experiences of women of low socioeconomic status regarding having children and contraceptive methods.

**Methods:**

A phenomenological design was adopted as a qualitative research method. It was conducted with 20 women living in a city in the Eastern Anatolia Region of Türkiye, with low socioeconomic status, married, and having at least one child, between March 15, 2023, and October 18, 2023. The data obtained were analyzed using the thematic content analysis method with the assistance of the Nvivo 12 Pro software package. COREQ guidelines were followed when reporting the data.

**Results:**

Analysis revealed three main themes: “Experiences regarding having children,” “Perceptions regarding having children,” and “Perceptions and experiences of contraceptive methods.” Most participants described having children as a sacred and meaningful experience yet emphasized the challenging aspects of this experience due to caregiving responsibilities and economic hardships. While the ideal number of children was generally stated as two, the actual number of children often exceeded this ideal; moreover, some women perceived the number of children as a matter of "destiny." Regarding contraceptive methods, lack of knowledge, the necessity of spousal approval, and concerns about side effects of certain methods were identified as key factors influencing participants’ contraceptive choices.

**Conclusion:**

The study revealed that complex emotional, relational, and social dynamics shape women’s decisions regarding childbearing. Additionally, knowledge gaps play a significant role in shaping their perceptions and use of contraceptive methods.

## What does this study add to the clinical work

**Table:** 

Lack of knowledge and economic difficulties in accessing contraceptive methods are important indicators of unintended pregnancies. Increasing women's level of education and encouraging their participation in the labor force can help them take greater ownership of their health and reproductive rights.	

## Background

In both developed and developing countries today, decisions regarding childbearing are directly influenced by fluctuations in economic conditions. While births are often postponed during times of financial crisis, they tend to accelerate during periods of stable economic growth [1]. The labor market flexibility brought about by globalization, increasing income inequalities, and growing social uncertainties negatively affect individuals’ living conditions, delaying decisions about having children [2]. In addition to these economic dynamics, factors such as climate change, migration, crime, wars, social instability, and individuals’ health conditions also play significant roles in shaping fertility decisions [1, 3].

These global trends affect countries worldwide, and fertility rates have declined to historically low levels not only in developed but also in developing nations. According to the Global Burden of Disease Study (2024), as of 2021, fertility rates in more than half of the world’s countries and regions have fallen below the population replacement level. Furthermore, future projections indicate that these rates are expected to remain low even if pronatalist policies are successfully implemented [4]. This suggests that the decline in fertility represents not a temporary fluctuation but a structural transformation. According to data from the Human Fertility Database, the average age at first birth in Türkiye is steadily increasing, while the average number of children per woman is decreasing [5]. Statistics from the Turkish Statistical Institute (TÜİK) reveal that the total fertility rate, 2.38 in 2001, fell to 1.51 in 2023, dropping below the population replacement level of 2.10. While fertility has declined nationwide in Türkiye during this period, the highest fertility rates continue to be concentrated in the Southeastern and Eastern Anatolia regions [6].

Alongside this global decline in fertility, inequalities in reproductive health also remain a pressing issue. According to the UNFPA’s 2022 report, approximately 121 million pregnancies occur annually, nearly half unintended. Expanding access to modern contraceptive methods has been one of the primary objectives of numerous international initiatives, including the Millennium Development Goals, Every Woman Every Child, Family Planning 2030 (FP2030), and the Sustainable Development Goals (SDGs) for 2030 [7]. Analyses conducted within the scope of FP2030 highlight that, although socio-economic inequalities in family planning have decreased over the past 30 years, supporting marginalized and vulnerable groups remains critically important [8].

Women’s inability to access modern contraceptive methods stems from multidimensional problems, including lack of information, social pressures, stigma, poverty, and the attitudes of healthcare providers [7]. As a result, unintended pregnancies have increased, with approximately 60% ending in abortion and about 45% of those performed under unsafe conditions. Unsafe abortions account for 5–13% of maternal deaths worldwide (Baker et al., 2022). It is estimated that around 160 million women globally have an unmet need for family planning [9], with this need being particularly high in Türkiye’s Eastern Anatolia region and among women with lower levels of education [10].

This study aims to conduct an in-depth examination of the perceptions and experiences of women with low socio-economic status regarding childbearing and contraceptive methods in a province located in Eastern Türkiye, where fertility rates remain high. In this context, the study is expected to contribute to a better understanding of existing reproductive health needs and inequalities in light of the social, cultural, and structural challenges experienced by these women.

## Methods

### Purpose

This study aims to conduct an in-depth examination of the perceptions and experiences of women with low socio-economic status regarding having children and contraceptive methods.

### Type of the study

This research was conducted using a qualitative research method in a phenomenological design through semi-structured individual interviews with women who reside in a province in the Eastern Anatolia Region of Türkiye, have low socio-economic status, are married, and have at least one child. This research design was preferred due to its ability to provide deep insights into the experiences of the individuals [11]. The Consolidated Criteria for Reporting Qualitative Research (COREQ) checklist guided the study.

### Population and sample

The population of this study consists of married women with low socio-economic status, having at least one child, residing in the province of Ağrı. The sample was determined based on the qualitative research design, ensuring diversity among participants and focusing on their expressed experiences. While there is no specific rule for sample size in phenomenological research, having a large sample is generally not recommended, as increasing the number of participants may risk losing the depth of data. “Saturation” is an important sample size guideline [12]. Therefore, data collection in our study was terminated when saturation was reached and data began to repeat. A total of 20 female participants who met the inclusion criteria—being married, having at least one child, residing in different regions of Ağrı, and not having any impediment to having more children—were interviewed.

### Data collection

The data collection process was conducted by a researcher with a doctoral degree in obstetrics and gynecology nursing and the Registered Nurse/Assistant Professor (FB) title. The researcher has received training and courses on qualitative research methods and is experienced in qualitative approaches. The data collection tool consisted of two parts: the first included eight questions to determine the socio-demographic characteristics of the women, and the second consisted of eight open-ended questions prepared by the scientific literature on childbearing and contraceptive methods. To ensure the data collection tool’s credibility and content validity, three experts in obstetrics and gynecology nursing and qualitative research reviewed the semi-structured interview form. Their feedback was used to assess the questions’ clarity, relevance, and appropriateness. Based on expert feedback, minor adjustments were made to improve the clarity and comprehensibility of the questions. These adjustments did not alter the content of the questions but were intended to enhance their clarity and ensure participants easily understood them. Sample questions included: (1) What are your thoughts about having children? (2) What do you think about the number of children? Who should decide on the number of children? (3) How did you decide on your pregnancies and having children? (4) What contraceptive method(s) have you used so far? (5) Where did you receive information about contraceptive methods? What are your thoughts and experiences regarding the methods? (6) Are you or your partner currently using any form of contraception? Why did you choose this method? (7) Would you like to have more children in the future? Why? At the end of the semi-structured questions, participants were also asked if there was anything else they wanted to add on any topic or thought in addition to the previous questions. Additionally, the data collection tool was evaluated through three pilot interviews conducted by the researcher to test the questions’ clarity, comprehensibility, and appropriateness. No further changes were necessary based on the pilot test results. The data obtained from the pilot interviews were not included in the final analysis.

The researchers followed Lincoln and Guba’s criteria for qualitative research rigor to enhance trustworthiness [13]. Credibility was ensured through prolonged engagement in the field and by conducting the interviews in participants’ familiar environments (their homes or health centers) to increase comfort and openness. Dependability was achieved by using the same semi-structured interview guide for all participants and recording all interviews with participant consent. Confirmability was supported by keeping detailed field notes and interview records, and transferability was addressed by providing detailed descriptions of the study context, participants, and data collection process. The research reached its aim when 20 participants were reached and the data collection process was completed. The interviews were conducted face-to-face by FB (Ph.D., Asst. Prof. Dr.), who has experience in qualitative interviewing in the participants’ homes or health centers at a time and place when they were alone. After obtaining their approval, the interviews were recorded with the participants’ consent using a voice recorder.

### Data analysis

The obtained data was checked for consistency with the audio recordings after transcription. The data were evaluated using the thematic content analysis method by Braun and Clarke (2006) in the Nvivo 12 Pro software package [14]. The following steps were followed in the data analysis process: (1) Familiarization with the data, (2) Generation of initial codes, (3) Searching for themes, (4) Reviewing themes, (5) Defining and naming themes, (6) Producing the report. The texts were read multiple times to understand the participants’ experiences holistically, and coding was independently conducted by researcher named (Ph.D., Asst. Prof. Dr.). Differences between the researchers were discussed, and adjustments were made until a consensus was reached on the themes. The results were presented as themes, sub-themes, and codes (Table 2). Throughout all the analysis steps, care was taken to ensure the reflection and fidelity of the narratives. Participant numbers were used instead of their real names.

### Ethical considerations

The study was conducted after obtaining ethical approval from the Ağrı İbrahim Çeçen University Scientific Research Ethics Committee, with approval number 198 dated October 10, 2022. All participants were informed about the purpose of the study, and it was emphasized that their participation was voluntary, they had the right to withdraw at any time, and their participation would be kept confidential. At the beginning of the research, participants’ verbal consent was recorded.

### Rigor

In this qualitative study, various approaches were employed to ensure reliability. Firstly, participant diversity was ensured using the intentional sampling method. Detailed and rich explanations were provided regarding the data collection procedures, and the data collection tool was subjected to pilot testing. Clear and detailed descriptions of the data analysis procedures were provided, and the researcher engaged in prolonged interaction with the data. Participant views and explanations were faithfully adhered to using direct quotations. Coding, sub-themes, and themes were evaluated multiple times by researchers experienced in qualitative research. The inter-coder agreement was determined to be 89%. The COREQ guidelines were utilized to report the data. Additionally, the researchers will keep all records confidential for three years. These approaches aim to enhance the reliability of the study in terms of validity, reliability, confirmability, transferability, and transparency.

## Results

### Participant characteristics

Interviews were conducted with 20 female participants in the study. The descriptive characteristics of the participants are presented in Table 1. All participants are female, with an average age of 31.60 ± 7.64 (minimum 21.00–maximum 48.00). While 25.0% of women (n = 5) are illiterate, 40.0% (n = 8) have completed primary school or below. Only one woman works as a cleaning staff; others do not have any income-generating employment. Women have experienced an average of 5.15 ± 2.30 (minimum 2.00–maximum 10.00) pregnancies and have an average of 3.95 ± 1.63 (minimum 2.00–maximum 9.00) children. The average age at first marriage for women is determined to be 18.45 ± 2.39 (minimum 14.00–maximum 24.00). As a contraceptive method, 45.0% of women (n = 9) use IUD, 30.0% (n = 6) practice withdrawal, 15.0% (n = 3) have undergone tubal ligation, and 5.0% (n = 1) use condoms (Table 1).

**Table 1:** Participant characteristics

Participants	Interview duration (min)	Age	Education level	Income status	Occupation	Family type	Number of children	Total number of pregnancies	Age at first marriage	Contraceptive method used
P 1	5.13	26	Middle School	Average	Housewife	Extended family	4	6	18	Tubal ligation
P 2	9.58	34	Illiterate	Poor	Housewife	Nuclear family	4	6	18	Tubal ligation
P 3	9.08	32	Associate’s Degree	Average	Housewife	Extended family	3	3	24	Intrauterine Device
P 4	9.54	33	Illiterate	Average	Housewife	Nuclear family	6	6	17	Intrauterine Device
P 5	8.02	40	Primary School	Average	Housewife	Nuclear family	4	8	16	Intrauterine Device
P 6	9.30	42	Primary School	Poor	Housewife	Nuclear family	6	10	17	Intrauterine Device
P 7	8.57	30	Primary School	Poor	Housewife	Nuclear family	3	5	22	Withdrawal
P 8	9.33	21	Middle School	Poor	Housewife	Extended family	4	6	14	Withdrawal
P 9	8.52	28	University	Poor	Housewife	Nuclear family	2	2	20	Intrauterine Device
P 10	6.30	48	Illiterate	Average	Housewife	Nuclear family	4	6	20	Not used
P 11	9.15	26	Literate	Poor	Housewife	Nuclear family	2	5	18	Intrauterine Device
P 12	7.52	38	Illiterate	Poor	Housewife	Nuclear family	5	6	22	Withdrawal
P 13	9.33	29	Middle School	Average	Cleaning Staff	Nuclear family	4	5	17	Tubal ligation
P 14	5.12	34	Primary School	Average	Housewife	Nuclear family	2	2	19	Condom
P 15	7.11	31	Primary School	Poor	Housewife	Nuclear family	4	4	20	Intrauterine Device
P 16	9.47	23	Middle School	Poor	Housewife	Extended family	3	3	15	Withdrawal
P 17	9.54	24	High School	Average	Housewife	Nuclear family	3	3	18	Intrauterine Device
P 18	7.02	21	Primary School	Poor	Housewife	Extended family	3	3	18	Withdrawal
P 19	9.00	28	Primary School	Average	Housewife	Extended family	4	4	18	Withdrawal
P 20	9.10	44	Illiterate	Poor	Housewife	Nuclear family	9	10	18	Intrauterine Device

During the interviews, each participant shared their thoughts on childbirth and contraception based on their life circumstances and experiences. The synthesis of the interviews resulted in three main themes: experiences related to childbirth, perceptions about childbirth, and perceptions and experiences related to contraceptive methods (Table 2).

**Table 2:** Main themes, analytic themes, and descriptive themes

Main theme	Analytic theme	Descriptive theme
Experiences regarding having children	Meanings attributed to children	Love, affection A miracle of God God-given Heart, soul A very beautiful feeling The world The person who will take care of the parents The joy of the house Future Life Everything Effort Happiness-Peace
	Difficulties experienced in childcare	Decreased tolerance Challenges of the era Economic inadequacies Lack of trust in the environment Failure to show enough attention Perceived as a woman’s duty
	Barriers to having children	Economic inadequacy Not wanting to sustain one’s own fate Health conditions not permitting Difficult living conditions
Perceptions regarding having children	Thoughts on the number of children	Should be few Should be both boys and girls (Gender) Destiny
	Decision-makers regarding having children	Mother Father Spouses Elder family members
Perceptions and experiences of contraceptive methods	Source of contraceptive information	Pharmacy Family doctor Nurse Environment Internet
	Challenges and obstacles in contraceptive method use	Ignorance Gender inequality Low educational level Economic inadequacy Lack of trust in men Unwanted pregnancies Dissatisfaction Opposition to modern methods
	Most preferred contraceptive methods in the region	Intrauterine device Withdrawal method Tubal ligation

#### Main theme 1. Experiences regarding having children

The analytical themes of the main theme regarding experiences of having children were determined as the meaning attributed to children, difficulties experienced in childcare, and barriers to having children.

##### Analytic theme 1.1. Meanings attributed to children

The majority of participants have attributed positive emotions to becoming parents. They emphasized that after becoming a mother, they experienced their most special and meaningful feelings, expressing that they would not trade their children for anything and hold onto life more tightly because of them.

*"Honestly, it is the most beautiful thing in the world. My children are everything to me… Right now, this child of mine is my most beautiful thing, my apple of the eye. I love him/her so much."* (P-16).

*"A child is something very different; a child becomes superior to everyone and everything else. I was very attached to my mother; I always wished nothing would happen to her. However, after having a child, the attachment to the mother ends. When you have a child, you do not care about anything happening in the world as long as your child does not bleed a drop of blood. Having a child is something different; I understood that when I became a mother."* (P-9).

*"Happiness, joy, attaching to life, experiencing motherhood. Everything becomes better with children. You have hope. I realized that there is someone you can share things with, someone you can trust."* (P-14).

Some participants, beyond positive emotions, associate having children with their religious beliefs, viewing it as a superior event, seeing it as a gift and miracle from God.

*"It’s given by God. It’s not something that anyone can control."* (P-5).

*"I see it as a miracle from God."* (P-3).

It was observed that some participants, beyond positive emotions and religious beliefs, see having children as a responsibility and perceive it as the person who will take care of them when they get older.

*"When you have children at home, for example, you deal with them. When they grow up, they have their schools. Their engagements, marriages… You deal with it."* (P-10).

*"At least let them grow up a bit so that they can take care of us in the future."* (P-17).

##### Analytic theme 1.2. Difficulties experienced in childcare

Participants experienced difficulties in childcare due to economic, environmental, social, and societal reasons. It was observed that economic inadequacies in the region deeply affected mothers in childcare and made it difficult, leading them not to want to have more children.

*"My little son is going to school too. For example, I did not send him to preschool this year because I did not pay for his transportation. He will go to first grade next year."* (P-7).

*"Especially when you go to villages… When people go to villages, it is a sin to see children barefoot, always in the dirt. No shoes on their feet. Plastic shoes, rubber shoes. Those who have it all live a rosy life, but those who do not are always in a mess."* (P-5).

*"When everything becomes so expensive, naturally, people have difficulties. That is why I had three children. I had a cesarean after the third one. They need a future. They need to be taken care of. Bringing them into the world is not a skill. What matters is taking good care of them. What matters is not neglecting their needs. What is the point if you bring them but do not do that?"* (P-7).

It was determined that childcare is perceived solely as the woman’s duty socially, and due to the lack of support from men, female participants are greatly exhausted and struggling during the childcare process. Additionally, they expressed that they are not allowed to meet their own needs because they are women.

*"…they do not let us go out, to take care of our own needs. They bring them themselves. We cannot go out, I mean."* (P-16).

*"I take care of the child. I raise him/her, I endure it, but my husband is not here. Since my child was born, he/she has been constantly admitted to the intensive care unit. He/she gets pneumonia and cannot breathe… My child is behind in everything. Neither can he/she sit nor move… I endure that hardship. My husband does not."* (P-16).

*"A child wears you out. The woman brings him/her up. The man says, ‘I earn, I bring it.’ However, that is not the case. The woman bears all the hardship. My husband goes to work (out of town) after the child turns forty days old. He does not see how I raised him/her, how I took care of him/her. I endure all the suffering… Fathers sleep, mothers wake up at night to breastfeed, and their sleep gets interrupted again, but the father does not have such a problem. When the father comes home from work, he says, ‘Is there anything to caring for a child?’… It is not just about feeding and putting to sleep. When a child gets sick, the mother gets worn out. When it comes to separation to the extent that Allah forbids, fathers say the child belongs to them."* (P-15).

Some participants mentioned that due to the gender roles and responsibilities imposed on women by society, their tolerance for taking care of children has decreased.

*"…One gets tired and cannot handle it anymore. Sometimes they fight, and I sit down and cry by myself. They do not stop, and children misbehave a bit."* (P-4).

##### Analytic theme 1.3. Barriers to having children

Participants’ reluctance to have more children is largely influenced by negative health conditions, economic inadequacies, the unwillingness to subject their children to their fate, and the challenging living conditions. It is observed that economic inadequacy is a significant factor affecting various aspects of participants’ lives, and they prefer to have a few children to avoid subjecting them to the difficulties they experienced during their childhood.

*"Let them have a life. At least we could not study. At least let our children study. At least he (the father) says let us not subject our children to that. I want them to have less but have everything."* (P-19).

*"…not because of taking care of children, but if the financial situation is inadequate, two children are enough, in my opinion. For the third, fourth… Since all their needs are not fully met, you feel sorry for yourself. You say, ‘I wish I had not brought them, then my children would not be lacking in everything like us."* (P-15).

*"Six pregnancies, three miscarriages, and I had one abortion. But I regretted it a lot. I aborted it because I did not have the financial means. Then I regretted it a lot."* (P-6).

#### Main theme 2. Perceptions regarding having children

The analytical themes of the main theme, "Perceptions regarding having children," were identified as thoughts about the number of children and determinants of deciding to have children.

##### Analytic theme 2.1. Thoughts on the number of children

In our study, although most participants expressed that the number of children should be few (usually 2), it was determined that the number of children is generally more than two. In some participants, beyond the number of children, gender was observed to be an important factor, and they expressed the number of their children as fate.

*"We actually thought of three. We always wanted three. Our fate is four. Enough for us. Actually, we always dreamed of three children."* (P-18).

*"At least when there are two children, a person can provide for both, take care of them financially. I buy something for one, the other one feels left out. It hurts me. I wish I had that child too."* (P-16).

*"Thank God we have two daughters and two sons. That is enough. Since there is no other gender, it is enough."* (P-13).

*"If God willing, if there are three girls, I want one boy. For example, if there are three boys, I want one girl. I want there to be a difference in at least one of them."* (P-17).

##### Analytic theme 2.2. Decision-makers regarding having children

It was observed that the determinants of having children vary, with men usually having more influence due to the patriarchal structure of society. However, most participants expressed that the decision-maker should be the mother.

*"I think the mother should make the decision. Because she takes care of everything. The one who takes all the worries, troubles… I mean, the woman is the one who even takes the husband’s nerves and his system. In my opinion, the decision for the child should be made by the woman."* (P-19).

*"For example, after the third child, I do not want more, but if it were up to the father, we would form a football team. I mean, his idea is that we will form a football team. We will have six. I do not want it, but I can do nothing."* (P-17).

*"…My husband decided on the fourth child. Well, that is how we completed it up to four. I am not thinking about it anymore, but my husband wants it."* (P-15).

#### Main theme 3. Perceptions and experiences of contraceptive methods

The analytical themes of the main theme of perceptions and experiences regarding contraceptive methods were identified as the source of contraceptive information, challenges and obstacles in contraceptive method use, and the most preferred contraceptive methods in the region.

##### Analytic theme 3.1. Source of contraceptive information

Most participants identified their sources of contraceptive information as doctors and nurses at health centers and their social environment, with very few mentioning pharmacies and the Internet.

*“I read about it on the internet. I research about whichever method I choose. There is even something called the subdermal capsule. I look into all of them.”* (P-13).

*“No one, absolutely no one. Doctors and nurses were the ones explaining it. I mean, at the health center.”* (P-5).

*“I heard about it from other women. From people around me.”* (P-4).

##### Analytic theme 3.2. Challenges and obstacles in contraceptive method use

Among the participants, it was found that ignorance about contraceptive methods is a significant issue, leading to a high rate of dissatisfaction due to unwanted pregnancies and contraceptive method failures.

*"I tried to use a pill at one point. I could not manage it. That is why I got pregnant."* (P-6).

*"…I don’t trust pills. Because people around me got pregnant when they used medication."* (P-18).

*"I got pregnant even though I was using an IUD myself, so I cannot say anything about another person’s experience."* (P-2).

It was observed that economic insufficiency is a significant problem in accessing and using contraceptive methods, leading to unwanted pregnancies.

*"I was happy with the pill. I would have continued with my pill if I had the means. But I could not continue due to economic reasons."* (P-1).

*"I used to take pills before. We used to get the pills from the health center. However, at some point, the Ministry stopped sending them. Our financial situation was not good then, so we could not afford it. Sometimes we could buy them, sometimes we could not. That is how we ended up getting pregnant."* (P-13).

Some participants faced challenges in accessing contraceptive methods due to gender inequality.

*"I am thinking of getting an IUD. My husband says no. I told my father-in-law to take me, but he did not. I asked them to take me. They said they would not interfere. They say your husband should come. My husband says no, I will not do it."* (P-16).

*"I cannot go alone. My mother-in-law says your husband is not here; please wait."* (P-17).

##### Analytic theme 3.3. Most preferred contraceptive methods in the region

It was determined that the most commonly used contraceptive methods among the participants were IUD, withdrawal, and tubal ligation. Despite experiencing many side effects with IUD, it was found to be the most preferred method by women.

*"I had much bleeding. I used the IUD for 9 years. I could not fast. I fasted for 15 days, and then I had to bleed for 15 days. I don’t know; the IUD was suitable for some people but not for others. In my opinion, the IUD is not good."* (P-20).

*"It constantly causes bleeding. You cannot do heavy work. You cannot clean properly. I mean, it constantly causes bleeding. It did not work for me… My legs still ache because of the IUD. It also caused a lot of pain in my heels."* (P-19).

Additionally, it was observed that after experiencing unwanted pregnancies following IUD usage, many participants considered tubal ligation.

*"Actually, if I were older, I would have my tubes tied because I do not want to have more children, but I am 21 years old, and the doctor says they will not do it, and so on."* (P-18).

*"…we kept getting pregnant. Now, I am thinking about getting my tubes tied. Hopefully, it will happen."* (P-13).

*"I actually want to get my tubes tied, sir. Because according to our situation, we cannot afford it. You know, everything has become expensive. We cannot afford it."* (P-2).

## Discussion

In this study, the perceptions and experiences of women with low socio-economic status regarding childbirth and contraceptive methods have been examined. As a result of the analysis of the interviews, three main themes were identified: “Experiences regarding having children,” “Perceptions regarding having children,” and “Perceptions and experiences regarding contraceptive methods”.

The analytical themes of the main theme of women’s experiences regarding having children were identified as “Meaning attributed to children,” “Difficulties experienced in childcare,” and "Barriers to having children.” Our study found that participants generally associate childbirth with positive and special emotions such as “love, affection, life, world, peace, happiness.” This finding is supported by previous qualitative studies, which have shown that children contribute positively to the emotional well-being of families and lead to a sharp increase in the psychological values attributed to children [15–17]. Overall, it is believed that children have positive psychological effects on parents and that this could be an influential factor in deciding to have children again.

In our study, it was determined that factors such as economic inadequacy, increasing difficulty of living conditions, and gender inequality pose obstacles for women in childcare and the process of having children again. Previous studies have also shown that women face difficulties in childcare and having children again due to economic inadequacy and the societal roles imposed on women [18, 19]. Our research, conducted with women of low socio-economic status, indicates that the economy negatively affects women’s decisions to have children and continue this process. Additionally, it is believed that gender inequality exacerbates the difficulties faced by women.

Perceptions regarding childbirth have been identified as the main theme of the study, with analytical themes including “Thoughts on the number of children” and “Decision-makers regarding having children.” Real, desired, and ideal numbers of children are commonly used in research to understand and predict fertility behavior in society [20]. In our study, participants generally expressed the ideal number of children as two. In contrast, for some participants, gender was an important factor beyond the number of children, and they referred to having more children as “destiny” according to their beliefs. Previous studies have shown that the importance given to having a male child is greater, religious beliefs play a significant role in the number of children, and the desired number of children is often fewer than the actual number of children [18, 21]. Factors such as the continuity of lineage, economic benefits, and patriarchal family structure are particularly influential in having male children. It is believed that women prefer to have fewer children due to the declining fertility rate. However, they end up having more children than the desired and preferred number due to the high rate of unwanted pregnancies among participants. Additionally, the transition from extended families to nuclear families in Turkish society, along with women entering or wanting to enter the workforce, is predicted to contribute to a decrease in the desired number of children.

Gender inequality is a significant factor influencing women’s decisions to have children [22]. In our study, it was found that decision-makers are generally men. When the literature is examined, it is observed that women have more decision-making power in developed countries [22, 23]; however, in underdeveloped or developing countries, men tend to be the primary decision-makers [18]. Due to the patriarchal structure of society and the roles assigned to women, women often take a backseat in decision-making regarding childbirth, and they are frequently not given a say even about their own bodies. This situation highlights the impact of gender inequality on the process of childbirth for women.

The analytical themes of the main theme concerning participants’ perceptions and experiences of contraceptive methods are as follows: “Source of contraceptive information,” “Challenges and obstacles in contraceptive method use,” and “Most preferred contraceptive methods in the region.” In our study, it was found that participants generally rely on doctors, nurses, and acquaintances at family health centers as sources of contraceptive information. However, very few participants found pharmacies and the Internet to be preferred. Previous studies have also observed that healthcare providers play a significant role as sources of information, followed by friends and family members, with pharmacies surprisingly being less preferred as a source of information [24–26]. It is speculated that due to the low economic status of the participants, they prefer healthcare providers at family health centers more, as they are free, and trust is also a factor. At the same time, pharmacies are less preferred due to being fee-based. Additionally, considering their low educational and economic levels, limited access to smartphones, the internet, and computers may hinder obtaining information online.

Our study findings indicate that lack of knowledge is the most significant barrier to contraceptive use, leading to issues in contraceptive method preference and utilization, consequently resulting in high rates of unintended pregnancies among participants. Particularly, research conducted in rural areas suggests that a lack of knowledge about contraceptive methods is widespread among women, posing a series of challenges for them [26, 27]. Regional and socioeconomic factors significantly influence women’s access to and attitudes toward contraceptive methods, often leading to unintended pregnancies that negatively impact maternal and fetal health. The most commonly used methods among participants were IUDs, withdrawal, and tubal ligation. Economic constraints drive preference for cost-effective (IUDs, tubal ligation) and easily accessible traditional methods (withdrawal). Despite high dissatisfaction, IUDs remained the most widely used. Withdrawal, despite its high failure rate, was the second most preferred, followed by tubal ligation among women no longer wishing to have children.

## Conclusion and recommendations

The study concluded that participants deeply value having children, viewing them as symbols of love, hope, and life’s purpose. Despite this, they face significant challenges such as childcare difficulties and economic constraints. Views on family size vary, influenced by personal preferences and partner input. Cultural and religious norms also shape perceptions of parenthood. Family health centers are the main source of information on contraception, yet users often face issues like lack of knowledge, financial barriers, and unintended pregnancies. The most common methods used are IUDs, withdrawal, and tubal ligation. To improve reproductive health access for women with low socioeconomic status, public health education should be expanded, including counseling by healthcare professionals. Promoting women’s education and employment can enhance their ability to exercise reproductive rights. Campaigns challenging traditional gender roles and advocating for gender equality should be strengthened.

## Data Availability

No datasets were generated or analysed during the current study.
